# Prognostic value and predication model of microvascular invasion in patients with intrahepatic cholangiocarcinoma: a multicenter study from China

**DOI:** 10.1186/s12885-021-09035-5

**Published:** 2021-12-05

**Authors:** Yifan Chen, Hongzhi Liu, Jinyu Zhang, Yijun Wu, Weiping Zhou, Zhangjun Cheng, Jianying Lou, Shuguo Zheng, Xinyu Bi, Jianming Wang, Wei Guo, Fuyu Li, Jian Wang, Yamin Zheng, Jingdong Li, Shi Cheng, Yongyi Zeng, Jingfeng Liu

**Affiliations:** 1 Department of Hepatopancreatobiliary Surgery, Mengchao Hepatobiliary Hospital of Fujian Medical University, Xihong Road 312, Fuzhou, 350025 Fujian Province People’s Republic of China; 2 Department of Hepatobiliary Surgery III, Eastern Hepatobiliary Surgery Hospital, Secondary Military Medical University, Shanghai, China; 3 Department of Hepatobiliary Surgery, The Affiliated Zhongda Hospital of Southeast University, Nanjing, China; 4 Department of Hepatobiliary Surgery, The Second Hospital Affiliated to Zhejiang University, Hangzhou, China; 5 Department of Hepatobiliary Surgery, The Southwest Hospital Affiliated to the Army Medical University, Chongqing, China; 6 Department of Hepatobiliary Surgery, Cancer Hospital, Chinese Academy of Medical Sciences, Beijing, China; 7 Department of Hepatobiliary Surgery, Tongji Hospital Affiliated to Tongji Medical College, Huazhong University of Science & Technology, Wuhan, China; 8 Department of Hepatobiliary Surgery, Beijing Friendship Hospital Affiliated to Capital Medical University, Beijing, China; 9 Department of Hepatobiliary Surgery, The West China Hospital of Sichuan University, Chengdu, China; 10 Department of Hepatobiliary Surgery, Renji Hospital Affiliated to Shanghai Jiaotong University, Shanghai, China; 11 Department of Hepatobiliary Surgery, Xuanwu Hospital Affiliated to Capital Medical University, Beijing, China; 12 Department of Hepatobiliary Surgery, The Affiliated Hospital of Chuanbei Medical University, Nanchong, China; 13 Department of Hepatobiliary Surgery, Tiantan Hospital Affiliated to Capital Medical University, Beijing, China; 14 Liver Diseases Center, The First Affiliated Hospital of Fujian Medical University, Fuzhou, China

**Keywords:** Intrahepatic cholangiocarcinoma, Microvascular invasion, Prognosis, Prediction model

## Abstract

**Background:**

At present, hepatectomy is still the most common and effective treatment method for intrahepatic cholangiocarcinoma (ICC) patients. However, the postoperative prognosis is poor. Therefore, the prognostic factors for these patients require further exploration. Whether microvascular invasion (MVI) plays a crucial role in the prognosis of ICC patients is still unclear. Moreover, few studies have focused on preoperative predictions of MVI in ICC patients.

**Methods:**

Clinicopathological data of 704 ICC patients after curative resection were retrospectively collected from 13 hospitals. Independent risk factors were identified by the Cox or logistic proportional hazards model. In addition, the survival curves of the MVI-positive and MVI-negative groups before and after matching were analyzed. Subsequently, 341 patients from a single center (Eastern Hepatobiliary Hospital) in the above multicenter retrospective cohort were used to construct a nomogram prediction model. Then, the model was evaluated by the index of concordance (C-Index) and the calibration curve.

**Results:**

After propensity score matching (PSM), Child-Pugh grade and MVI were independent risk factors for overall survival (OS) in ICC patients after curative resection. Major hepatectomy and MVI were independent risk factors for recurrence-free survival (RFS). The survival curves of OS and RFS before and after PSM in the MVI-positive groups were significantly different compared with those in the MVI-negative groups. Multivariate logistic regression results demonstrated that age, gamma-glutamyl transpeptidase (GGT), and preoperative image tumor number were independent risk factors for the occurrence of MVI. Furthermore, the prediction model in the form of a nomogram was constructed, which showed good prediction ability for both the training (C-index = 0.7622) and validation (C-index = 0.7591) groups, and the calibration curve showed good consistency with reality.

**Conclusion:**

MVI is an independent risk factor for the prognosis of ICC patients after curative resection. Age, GGT, and preoperative image tumor number were independent risk factors for the occurrence of MVI in ICC patients. The prediction model constructed further showed good predictive ability in both the training and validation groups with good consistency with reality.

## Introduction

According to the different anatomical positions, cholangiocarcinoma can be divided into distal cholangiocarcinoma, hilar cholangiocarcinoma, and intrahepatic cholangiocarcinoma (ICC), which originate from secondary and above bile duct branches [1]. Among them, ICC is a malignant tumor derived from intrahepatic bile duct epithelium cells, and its incidence has gradually increased in recent years, and it now accounts for approximately 5–20% of primary liver cancer [1, 2]. The incidence of ICC varies greatly around the world, while the incidence of ICC in China ranks third in the world [3]. Because the symptoms for the early stage of ICC patients are relatively atypical, most cases are found in the middle or late stages. Hepatectomy remains the most common and effective treatment for ICC at present [4]. However, the prognosis after hepatectomy is unsatisfactory, and the 5-years survival rate is reported to be approximately 20–40%. Factors related to prognosis have been reported, including tumor diameter, tumor number, serum carbohydrate antigen 199 (CA199) level, lymph node metastasis, large vascular invasion, and antiviral therapy [4–7].

Large vascular invasion has been included as one of the most important indicators for the staging system in the 8th edition of the ICC staging criteria of the American Joint Committee on Cancer (AJCC) [8]. Vascular invasion includes microvascular invasion (MVI) and large vascular invasion. MVI, also known as microvascular cancer thrombus, means that endovascular cancer cell nests can be found under microscopic examination, and are mainly distributed in the tumor-adjacent hepatic vein and portal vein [9]. A large number of studies have proven that in hepatocellular carcinoma (HCC), MVI is a crucial risk factor for its prognosis [10, 11]. In addition, MVI status could be an indicator for patients whether they need postoperative adjuvant treatment, including transhepatic arterial chemotherapy and embolization (TACE) and sorafenib [12]. However, relevant studies about MVI in ICC patients demonstrate different prognostic impacts. In a study that adopted the propensity score matching (PSM) method, Tang et al. showed that MVI was a vital factor for overall survival (OS) and recurrence-free survival (RFS) after hepatectomy [13]. Another multicenter study suggested that MVI was a crucial factor for RFS but not for OS [14]. Therefore, whether MVI plays an important role in the prognosis of ICC patients remains unclear.

At present, few studies have focused on the prediction of MVI in preoperative ICC patients. Only the correlation analysis between preoperative imaging features, clinical indicators and MVI has been reported [13, 15–17]. Knowing the MVI status would facilitate the adoption of more active treatment methods for high-risk patients, such as anatomical resection to expand the distance of the surgical margin and adjuvant therapies, including TACE, radiotherapy, and immunotherapy, to achieve a better prognosis. However, relevant reports on the benefits of active surgical treatments and postoperative adjuvant therapy are not available.

Based on the above situation, it is of great value to explore whether MVI is crucial for the prognosis of ICC patients after curative resection and identify relevant preoperative indicators for predicting MVI.

## Methods

### Patient selection

This study retrospectively collected clinicopathological data for 704 ICC patients who underwent curative resection from December 2009 to December 2017, including 13 medical units (Mengchao Hepatobiliary Hospital, Eastern Hepatobiliary Surgery Hospital, Affiliated Zhongda Hospital of Southeast University, Affiliated Second Hospital of Zhejiang University, Southwest Hospital, Affiliated Cancer Hospital of Chinese Academy of Medical Sciences, Tongji Hospital, Beijing Friendship Hospital, West China Hospital, Renji Hospital, Xuanwu Hospital, Affiliated Hospital of Chuanbei Medical University, and Tiantan Hospital).

Patients were enrolled in this research if they met the following inclusion criteria: (1) ICC was confirmed by postoperative pathology; (2) curative resection (R0 resection) was performed; (3) no history of preoperative antitumor therapy; and (4) no history of distant metastasis or large vascular invasion by preoperative evaluation. Patients who had a history of other malignant tumors, were confirmed to have recurrent ICC, had died or were lost to follow-up within 30 days after surgery were excluded.

Due to incomplete preoperative blood test and imaging data from multiple centers, the preoperative prediction model in this study only included the single center of Eastern Hepatobiliary Hospital from the abovementioned multicenter retrospective cohort patients for further analysis. The exclusion criteria were as follows: (1) incomplete preoperative blood test and imaging data; and (2) preoperative imaging suggesting large vascular invasion and lymph node metastasis. The remaining inclusion and exclusion criteria were the same as above.

Signed informed consent to the use of their clinical data for clinical analysis was obtained prior to surgery.

### Data collection

Collected indicators included general information (sex, age, history of hepatitis, preoperative Child-Pugh grade), preoperative blood test indices (total bilirubin, serum CA199, and serum carcinoembryonic antigen (CEA)), intraoperative situation (lymph node dissection, blood loss, blood transfusion, and surgery method), and pathological indicators (tumor diameter, tumor number, satellites, surgical margin, pathological differentiation, lymph node metastasis, and MVI status). The histopathology results of the surgically resected specimen were diagnosed by three pathologists. If there was any dispute, consensus was reached through internal discussion. Among the indicators, MVI was defined as nests of cancer cells found in the lumen lined with endothelial cells under microscopic examination after standard pathological sampling. R0 resection is defined as the surgical margin without gross or microscopic evidence of tumor invasion.

Data from patients in Eastern Hepatobiliary Hospital with complete preoperative data from the above multicenter retrospective cohort was used to construct the prediction model. Collected indices included general information, preoperative blood test indices (routine blood examination, total bilirubin, albumin, gamma-glutamyl transpeptidase (GGT), AFP, CA199, and CEA), and preoperative imaging data (tumor diameter and tumor number). Preoperative imaging data were extracted in the priority order of abdominal magnetic resonance imaging (MRI), computed tomography (CT), and abdominal ultrasound. Inflammatory indices were calculated according to the following formulas: NLR = neutrophil-to-lymphocyte ratio [18], PLR = platelet-to-lymphocyte ratio [19], and LMR = lymphocyte-to-monocyte ratio [20].

### Follow-up

According to the standard procedures, patients should be followed up once every 3 months for the first 2 years after surgery and every 3 to 6 months thereafter. During the follow-up program, routine monitoring examinations generally included medical history collection, blood sample tests, and imaging examinations. According to the actual condition of the patient, the clinician decided whether to perform further examinations, such as chest CT, brain CT, and radionuclide bone imaging, to rule out the possibility of recurrence and metastasis. The time interval between the date of surgery and death from any cause or the last follow-up was defined as OS. The period from the date of surgery to the first detection of recurrence or the last follow-up was defined as RFS. The primary outcome in this study was OS and RFS. The follow-up period was completed on 31 December 2020.

### Statistical analysis

R 4.0.2 and SPSS 25 were used for the statistical analyses. Chi-square or Fisher’s exact tests were adopted to compare categorical variables. The PSM method was adopted to minimize selection bias, matching at a ratio of 1:2 was performed using the nearest neighbor method, with a caliper value of 0.1. The Cox proportional hazards model was adopted to detect the independent factors of prognosis before and after PSM. Survival curves were analyzed by the Kaplan-Meier method using a log-rank test to compare the differences between groups.

Univariate and multivariate analyses of logistic regression models were used to analyze the MVI-related factors of ICC patients in the training group, and then a nomogram prediction model was constructed based on the multivariate results. The prediction accuracy of the model was estimated by the index of concordance (C-index). The consistency between the predicted results and the actual situation in the training and validation groups was evaluated by the calibration curves. Statistical results with *p* < 0.05 were considered statistically significant.

## Results

### Baseline characteristics

**Fig. 1:**
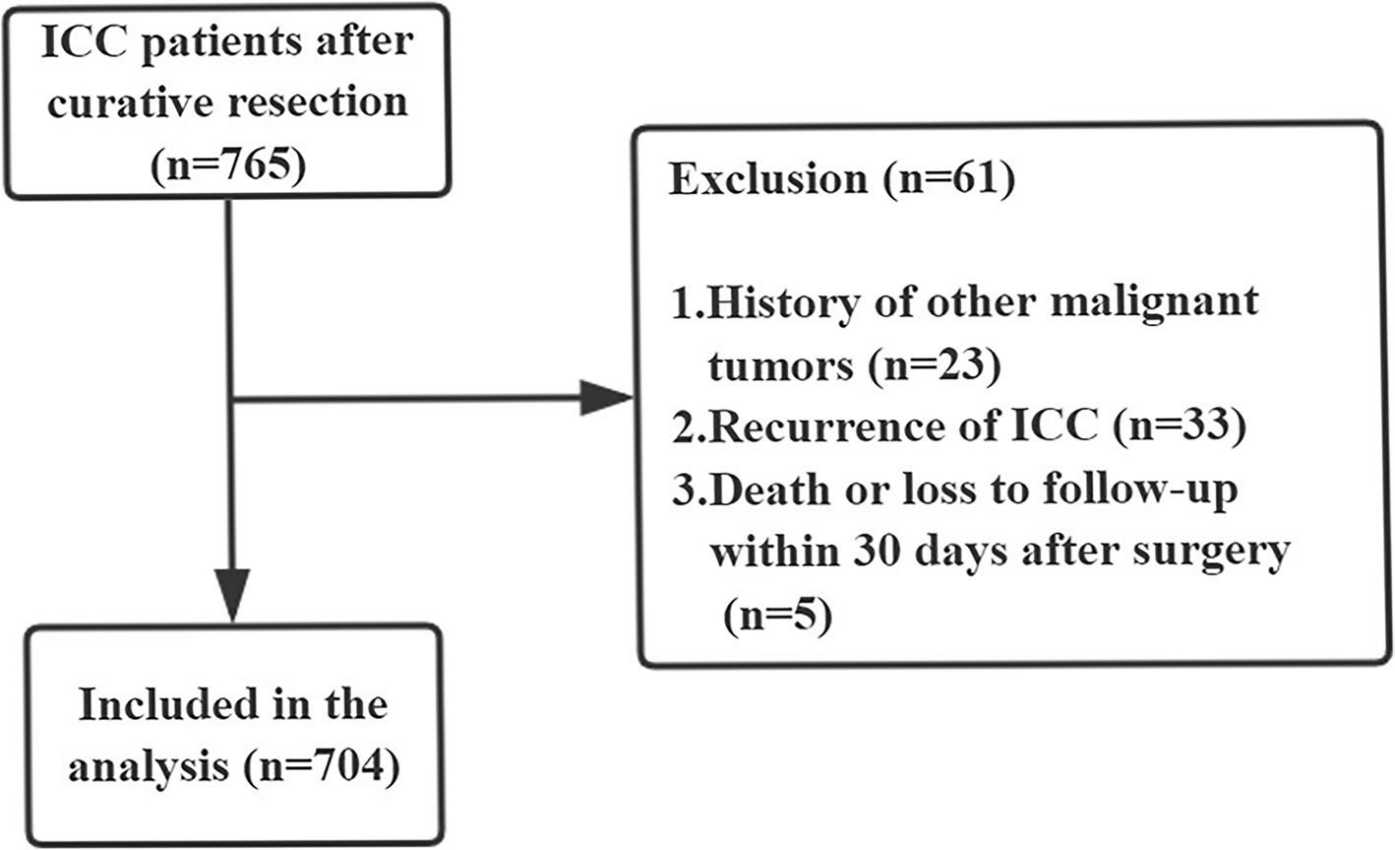
The flow chart of selected patients. ICC, Intrahepatic cholangiocarcinoma

A total of 765 ICC patients who underwent curative hepatectomy and met the inclusion criteria were initially taken into consideration. Subsequently, 61 patients were excluded, including 23 patients with a history of other malignant tumors, 33 patients diagnosed with recurrent ICC, and 5 patients who were lost to follow-up or died within 1 month after surgery. Finally, 704 patients were enrolled, and they consisted of 81 patients with MVI-positive status and 623 without (Fig. 1).

The baseline characteristics of all 704 patients and the comparison of the different MVI status groups are summarized in Table 1. The MVI-positive group had a worse Child-Pugh grade (*p* = 0.026) than the MVI-negative group, while the other general characteristics did not significantly differ. Considering the preoperative blood test indices, only CEA (*p* = 0.007) was higher in the MVI-positive group. More patients received lymph node dissection (*p* = 0.009) and major hepatectomy (*p* = 0.013) and had more blood loss (*p* = 0.009) during the surgery in the MVI positive group. Some pathologically related factors were significantly different between the groups, such as the tumor diameter (*p* = 0.045), tumor number (*p* = 0.046), pathological differentiation (*p* = 0.002), lymph node metastasis (*p* = 0.047), and satellites (*p* = 0.005).

**Table 1:** Comparison of clinicopathological characteristics between MVI-positive and negative group before PSM

Variables	MVI-negative group	MVI-positive group	***P-value***	Total
	(***N*** = 623)	(***N*** = 81)		(***N*** = 704)
**Age,** year				
< 60	369 (59.2%)	55 (67.9%)	0.168	424 (60.2%)
≥ 60	254 (40.8%)	26 (32.1%)		280 (39.8%)
**Sex**				
Male	365 (58.6%)	43 (53.1%)	0.410	408 (58.0%)
Female	258 (41.4%)	38 (46.9%)		296 (42.0%)
**Hepatitis**				
No	397 (63.7%)	54 (66.7%)	0.692	451 (64.1%)
Yes	226 (36.3%)	27 (33.3%)		253 (35.9%)
**Child-Pugh grade**				
Stage A	455 (73.0%)	69 (85.2%)	0.026	524 (74.4%)
Stage B	168 (27.0%)	12 (14.8%)		180 (25.6%)
**Total bilirubin**, μmol/L				
≤ 20	389 (62.4%)	49 (60.5%)	0.827	438 (62.2%)
> 20	234 (37.6%)	32 (39.5%)		266 (37.8%)
**CA199**, U/mL				
< 200	493 (79.1%)	60 (74.1%)	0.368	553 (78.6%)
≥ 200	130 (20.9%)	21 (25.9%)		151 (21.4%)
**CEA**, μg/L				
< 5	488 (78.3%)	52 (64.2%)	0.007	540 (76.7%)
≥ 5	135 (21.7%)	29 (35.8%)		164 (23.3%)
**Operative bleeding loss**, ml				
≥ 800	517 (83.0%)	57 (70.4%)	0.009	574 (81.5%)
< 800	106 (17.0%)	24 (29.6%)		130 (18.5%)
**Blood transfusion**				
No	527 (84.6%)	69 (85.2%)	1.000	596 (84.7%)
Yes	96 (15.4%)	12 (14.8%)		108 (15.3%)
**Laparoscopy**				
Yes	30 (4.8%)	5 (6.2%)	0.797	35 (5.0%)
No	593 (95.2%)	76 (93.8%)		669 (95.0%)
**Lymph node dissection**				
No	426 (68.4%)	43 (53.1%)	0.009	469 (66.6%)
Yes	197 (31.6%)	38 (46.9%)		235 (33.4%)
**Major hepatectomy**				
No	280 (44.9%)	24 (29.6%)	0.013	304 (43.2%)
Yes	343 (55.1%)	57 (70.4%)		400 (56.8%)
**Tumor diameter,** cm				
≤ 5	245 (39.3%)	22 (27.2%)	0.045	267 (37.9%)
> 5	378 (60.7%)	59 (72.8%)		437 (62.1%)
**Tumor number**				
Single	455 (73.0%)	50 (61.7%)	0.046	505 (71.7%)
Multiple	168 (27.0%)	31 (38.3%)		199 (28.3%)
**Satellite nodules**				
No	465 (74.6%)	48 (59.3%)	0.005	513 (72.9%)
Yes	158 (25.4%)	33 (40.7%)		191 (27.1%)
**Surgical margin width,** cm				
< 1	432 (69.3%)	52 (64.2%)	0.417	484 (68.8%)
≥ 1	191 (30.7%)	29 (35.8%)		220 (31.2%)
**Differentiation**				
Moderate or high	510 (81.9%)	54 (66.7%)	0.002	564 (80.1%)
Poor	113 (18.1%)	27 (33.3%)		140 (19.9%)
**Lymph node metastasis**				
No	533 (85.6%)	57 (70.4%)	< 0.001	590 (83.8%)
Yes	90 (14.4%)	24 (29.6%)		114 (16.2%)
**Hepatolith**				
No	602 (96.6%)	77 (95.1%)	0.691	679 (96.4%)
Yes	21 (3.4%)	4 (4.9%)		25 (3.6%)
**Postoperative hospital stay**, day				
< 15	366 (58.7%)	34 (42.0%)	0.006	400 (56.8%)
≥ 15	257 (41.3%)	47 (58.0%)		304 (43.2%)

*Abbreviations*: *MVI* Microvascular invasion, *PSM* Propensity Score Matching, *CA199* Carbohydrate antigen 199, *CEA* Carcinoembryonic antigen

Subsequently, PSM was performed for the MVI-positive group according to the above factors and matched at a ratio of 1:2, with a caliper value of 0.1. Unfortunately, 3 patients in the MVI-positive group were not successfully matched in the MVI-negative group. Among them, 5 patients in the MVI-positive group successfully matched only 1 patient in the MVI-negative group. Therefore, 229 patients were enrolled in the cohort after PSM, among which 78 patients were MVI positive and 151 were negative. The baseline characteristics did not show statistically significant difference between groups (*p* ≥ 0.05) (Table 2).

**Table 2:** Comparison of clinicopathological characteristics between MVI-positive and negative group after PSM

Variables	MVI-negative group	MVI-positive group	***P-value***	Total
	(***N*** = 151)	(***N*** = 78)		(***N*** = 229)
**Age,** year				
< 60	102 (67.5%)	52 (66.7%)	1.000	154 (67.2%)
≥ 60	49 (32.5%)	26 (33.3%)		75 (32.8%)
**Sex**				
Male	86 (57.0%)	42 (53.8%)	0.758	128 (55.9%)
Female	65 (43.0%)	36 (46.2%)		101 (44.1%)
**Hepatitis**				
No	94 (62.3%)	51 (65.4%)	0.748	145 (63.3%)
Yes	57 (37.7%)	27 (34.6%)		84 (36.7%)
**Child-Pugh grade**				
Stage A	132 (87.4%)	66 (84.6%)	0.701	198 (86.5%)
Stage B	19 (12.6%)	12 (15.4%)		31 (13.5%)
**Total bilirubin**, μmol/L				
≤ 20	95 (62.9%)	48 (61.5%)	0.952	143 (62.4%)
> 20	56 (37.1%)	30 (38.5%)		86 (37.6%)
**CA199**, U/mL				
< 200	111 (73.5%)	58 (74.4%)	1.000	169 (73.8%)
≥200	40 (26.5%)	20 (25.6%)		60 (26.2%)
**CEA**, μg/L				
< 5	100 (66.2%)	51 (65.4%)	1.000	151 (65.9%)
≥ 5	51 (33.8%)	27 (34.6%)		78 (34.1%)
**Operative bleeding loss**, ml				
≥ 800	118 (78.1%)	56 (71.8%)	0.367	174 (76.0%)
< 800	33 (21.9%)	22 (28.2%)		55 (24.0%)
**Blood transfusion**				
No	527 (84.6%)	69 (85.2%)	1.000	596 (84.7%)
Yes	96 (15.4%)	12 (14.8%)		108 (15.3%)
**Laparoscopy**				
Yes	142 (94.0%)	73 (93.6%)	1.000	215 (93.9%)
No	9 (6.0%)	5 (6.4%)		14 (6.1%)
**Lymph node dissection**				
No	80 (53.0%)	43 (55.1%)	0.866	123 (53.7%)
Yes	71 (47.0%)	35 (44.9%)		106 (46.3%)
**Major hepatectomy**				
No	48 (31.8%)	24 (30.8%)	0.994	72 (31.4%)
Yes	103 (68.2%)	54 (69.2%)		157 (68.6%)
**Tumor diameter,** cm				
≤ 5	43 (28.5%)	22 (28.2%)	1.000	65 (28.4%)
> 5	108 (71.5%)	56 (71.8%)		164 (71.6%)
**Tumor number**				
Single	88 (58.3%)	47 (60.3%)	0.883	135 (59.0%)
Multiple	63 (41.7%)	31 (39.7%)		94 (41.0%)
**Satellite nodules**				
No	89 (58.9%)	46 (59.0%)	1.000	135 (59.0%)
Yes	62 (41.1%)	32 (41.0%)		94 (41.0%)
**Surgical margin width,** cm				
< 1	107 (70.9%)	51 (65.4%)	0.485	158 (69.0%)
≥ 1	44 (29.1%)	27 (34.6%)		71 (31.0%)
**Differentiation**				
Moderate or high	108 (71.5%)	54 (69.2%)	0.835	162 (70.7%)
Poor	43 (28.5%)	24 (30.8%)		67 (29.3%)
**Lymph node metastasis**				
No	111 (73.5%)	57 (73.1%)	1.000	168 (73.4%)
Yes	40 (26.5%)	21 (26.9%)		61 (26.6%)
**Hepatolith**				
No	142 (94.0%)	74 (94.9%)	1.000	216 (94.3%)
Yes	9 (6.0%)	4 (5.1%)		13 (5.7%)
**Postoperative hospital stay**, day				
< 15	63 (41.7%)	34 (43.6%)	0.897	97 (42.4%)
≥ 15	88 (58.3%)	44 (56.4%)		132 (57.6%)

*Abbreviations*: *MVI* Microvascular invasion, *PSM* Propensity Score Matching, *CA199* Carbohydrate antigen 199, *CEA* Carcinoembryonic antigen

### Cox analysis for prognosis in ICC patients after curative resection

According to clinical experience and relevant previous studies, 19 observed indicators were finally included for analysis. Prior to PSM, the univariate analysis showed that the Child-Pugh grade, total bilirubin, CEA, tumor number, tumor diameter, surgical margin width, satellites, major hepatectomy, and MVI were associated with OS (*p* < 0.05). The Child-Pugh grade, total bilirubin, tumor number, tumor diameter, satellites, major hepatectomy, and MVI were correlated with RFS (*p* < 0.05). The above indicators at *p* < 0.05 were included in the multivariate analysis, and the stepwise forward method was adopted. The results demonstrated that the Child-Pugh grade (*p* = 0.006), total bilirubin (*p* = 0.014), CEA (*p* = 0.016), pathological tumor diameter (*p* < 0.001), satellites (*p* = 0.001), surgical margin width (*p* = 0.043), and MVI (*p* = 0.036) were independent risk factors for OS in ICC patients after curative resection. While Child-Pugh grade (*p* < 0.001), total bilirubin (*p* = 0.009), major hepatectomy (*p* < 0.001), satellites (*p* < 0.001), and MVI (*p* < 0.001) were independent risk factors for RFS (Tables 3 and 4).

**Table 3:** Univariate and multivariate Cox regression for overall survival in ICC patients after curative resection before PSM

Variables	Univariate			Multivariate		
	HR	95% CI	***p***-value	HR	95% CI	***p-value***
**Age**, year	1.114	0.900–1.379	0.319			
**Sex,** male vs female	1.065	0.861–1.317	0.563			
**Hepatitis**, no vs yes	0.926	0.742–1.156	0.498			
**Child-Pugh grade**, Grade A vs Grade B	1.404	1.122–1.757	0.003	1.394	1.098–1.769	0.006
**Total bilirubin,** μmol/L < 20 vs ≥20	1.346	1.089–1.663	0.006	1.320	1.057–1.648	0.014
**CA199,** U/mL < 200 vs ≥200	1.066	0.822–1.383	0.630			
**CEA,** μg/L < 5 vs ≥5	1.302	1.020–1.660	0.034	1.364	1.060–1.754	0.016
**Blood loss**, ml < 800 vs ≥800	1.162	0.890–1.518	0.270			
**Blood transfusion**, no vs yes	1.035	0.771–1.390	0.820			
**Tumor diameter,** cm ≤5 vs > 5	1.730	1.377–2.174	< 0.001	1.560	1.236–1.968	< 0.001
**Tumor number** single vs multiple	1.614	1.289–2.020	< 0.001			
**Surgical margin width,** cm < 1 vs ≥1	0.745	0.588–0.945	0.015	0.777	0.608–0.992	0.043
**Satellite nodules** no vs yes	1.689	1.350–2.112	< 0.001	1.487	1.175–1.882	0.001
**Laparoscopy** no vs yes	1.074	0.639–1.806	0.786			
**Differentiation** moderate or high vs poor	0.957	0.731–1.254	0.751			
**Major hepatectomy** no vs yes	1.734	1.387–2.169	< 0.001			
**Hepatolith** no vs yes	1.325	0.776–2.264	0.303			
**MVI** no vs yes	1.636	1.215–2.203	0.001	1.399	1.022–1.916	0.036
**Postoperative hospital stay**, day < 15 vs ≥15	1.111	0.898–1.374	0.335			

*Abbreviations*: *ICC* Intrahepatic cholangiocarcinoma, *PSM* Propensity Score Matching, *HR* Hazard ratio, *CI* Confidence interval, *CA199* Carbohydrate antigen 199, *CEA* Carcinoembryonic antigen, *MVI* Microvascular invasion

**Table 4:** Univariate and multivariate Cox regression for recurrence-free survival in ICC patients after curative resection before PSM

Variables	Univariate			Multivariate		
	HR	95% CI	***p***-value	HR	95% CI	***P-value***
**Age**, year	0.879	0.713–1.083	0.227			
**Sex,** male vs female	1.098	0.895–1.347	0.369			
**Hepatitis**, no vs yes	0.946	0.764–1.171	0.610			
**Child-Pugh grade**, Grade A vs Grade B	1.655	1.335–2.052	< 0.001	1.539	1.221–1.939	< 0.001
**Total bilirubin,** μmol/L < 20 vs ≥20	1.523	1.242–1.867	< 0.001	1.332	1.074–1.651	0.009
**CA199,** U/mL < 200 vs ≥200	0.798	0.614–1.036	0.090			
**CEA,** μg/L < 5 vs ≥5	0.983	0.768–1.257	0.889			
**Blood loss**, ml < 800 vs ≥800	1.065	0.821–1.382	0.634			
**Blood transfusion**, no vs yes	0.838	0.622–1.129	0.246			
**Tumor diameter,** cm ≤5 vs > 5	1.481	1.194–1.836	< 0.001			
**Tumor number** single vs multiple	1.440	1.155–1.796	0.001			
**Surgical margin width,** cm < 1 vs ≥1	0.851	0.682–1.062	0.154			
**Satellite nodules** no vs yes	1.686	1.357–2.094	< 0.001	1.511	1.206–1.893	< 0.001
**Laparoscopy** no vs yes	0.674	0.388–1.173	0.163			
**Differentiation** moderate or high vs poor	0.785	0.599–1.029	0.080			
**Major hepatectomy** no vs yes	1.815	1.465–2.249	< 0.001	1.535	1.231–1.915	< 0.001
**Hepatolith** no vs yes	0.990	0.569–1.722	0.971			
**MVI** no vs yes	1.840	1.392–2.431	< 0.001	1.742	1.303–2.329	< 0.001
**Postoperative hospital stay**, day < 15 vs ≥15	1.043	0.849–1.281	0.688			

*Abbreviations*: *ICC* Intrahepatic cholangiocarcinoma, *PSM* Propensity Score Matching, *HR* Hazard ratio, *CI* Confidence interval, *CA199* Carbohydrate antigen 199, *CEA* Carcinoembryonic antigen, *MVI* Microvascular invasion

After PSM, the univariate analysis showed that the Child-Pugh grade, pathological tumor diameter, major hepatectomy, and MVI were correlated with OS (*p* < 0.05). The preoperative Child-Pugh grade, total bilirubin, pathological tumor diameter, major hepatectomy, pathological differentiation, and MVI were associated with RFS (*p* < 0.05). Subsequently, the multivariate analysis demonstrated that the Child-Pugh grade (*p* = 0.001) and MVI (*p* = 0.014) were independent risk factors for OS in ICC patients after curative resection. Major hepatectomy (*p* = 0.001) and MVI (*p* < 0.001) were independent risk factors for RFS (Tables 5 and 6).

**Table 5:** Univariate and multivariate Cox regression for overall survival in ICC patients after curative resection after PSM

Variables	Univariate			Multivariate		
	HR	95% CI	***P***-value	HR	95% CI	***P-value***
**Age**, year	1.218	0.841–1.764	0.297			
**Sex,** male vs female	1.006	0.703–1.440	0.973			
**Hepatitis**, no vs yes	0.879	0.602–1.283	0.505			
**Child-Pugh grade**, Grade A vs Grade B	2.187	1.407–3.399	0.001	2.098	1.344–3.274	0.001
**Total bilirubin,** μmol/L < 20 vs ≥20	1.325	0.927–1.895	0.123			
**CA199,** U/mL < 200 vs ≥200	0.814	0.53–1.249	0.346			
**CEA,** μg/L < 5 vs ≥5	1.431	0.991–2.066	0.056			
**Blood loss**, ml < 800 vs ≥800	1.000	0.667–1.498	0.999			
**Blood transfusion**, no vs yes	0.798	0.477–1.333	0.388			
**Tumor diameter,** cm ≤5 vs > 5	1.529	1.006–2.323	0.047			
**Tumor number** single vs multiple	1.215	0.848–1.740	0.289			
**Surgical margin width,** cm < 1 vs ≥1	0.940	0.632–1.397	0.759			
**Satellite nodules** no vs yes	1.212	0.847–1.734	0.293			
**Laparoscopy** no vs yes	0.978	0.455–2.100	0.954			
**Differentiation** moderate or high vs poor	0.868	0.577–1.306	0.498			
**Major hepatectomy** no vs yes	1.526	1.014–2.297	0.043	1.423	0.941–2.15	0.094
**Hepatolith** no vs yes	1.213	0.592–2.486	0.598			
**MVI** no vs yes	1.542	1.073–2.217	0.019	1.576	1.096–2.266	0.014
**Postoperative hospital stay**, day < 15 vs ≥15	0.894	0.624–1.282	0.542			

*Abbreviations*: *ICC* Intrahepatic cholangiocarcinoma, *PSM* Propensity Score Matching, *HR* Hazard ratio, *CI* Confidence interval, *CA199* Carbohydrate antigen 199, *CEA* Carcinoembryonic antigen, *MVI* Microvascular invasion

**Table 6:** Univariate and multivariate Cox regression for recurrence-free survival in ICC patients after curative resection after PSM

Variables	Univariate			Multivariate		
	HR	95% CI	***P***-value	HR	95% CI	***P-value***
**Age**, year	1.047	0.723–1.517	0.807			
**Sex,** male vs female	1.192	0.840–1.692	0.326			
**Hepatitis**, no vs yes	0.899	0.620–1.302	0.572			
**Child-Pugh grade**, Grade A vs Grade B	2.004	1.273–3.153	0.003	1.601	0.991–2.586	0.055
**Total bilirubin,** μmol/L < 20 vs ≥20	1.489	1.049–2.113	0.026	1.321	0.915–1.907	0.137
**CA199,** U/mL < 200 vs ≥200	0.795	0.525–1.204	0.279			
**CEA,** μg/L < 5 vs ≥5	1.031	0.710–1.498	0.871			
**Blood loss**, ml < 800 vs ≥800	1.086	0.730–1.617	0.683			
**Blood transfusion**, no vs yes	0.771	0.462–1.286	0.319			
**Tumor diameter,** cm ≤5 vs > 5	1.650	1.089–2.501	0.018			
**Tumor number** single vs multiple	1.262	0.885–1.798	0.198			
**Surgical margin width,** cm < 1 vs ≥1	0.964	0.657–1.415	0.853			
**Satellite nodules** no vs yes	1.357	0.954–1.930	0.090			
**Laparoscopy** no vs yes	0.742	0.327–1.686	0.476			
**Differentiation** moderate or high vs poor	0.619	0.407–0.942	0.025	0.727	0.474–1.116	0.145
**Major hepatectomy** no vs yes	2.327	1.510–3.585	< 0.001	2.147	1.386–3.326	0.001
**Hepatolith** no vs yes	0.950	0.443–2.036	0.894			
**MVI** no vs yes	2.038	1.433–2.898	< 0.001	2.057	1.445–2.929	< 0.001
**Postoperative hospital stay**, day < 15 vs ≥15	1.022	0.715–1.460	0.905			

*Abbreviations*: *ICC* Intrahepatic cholangiocarcinoma, *PSM* Propensity Score Matching, *HR* Hazard ratio, *CI* Confidence interval, *CA199* Carbohydrate antigen 199, *CEA* Carcinoembryonic antigen, *MVI* Microvascular invasion

### Impact of MVI on the prognosis of ICC patients after curative resection

**Fig. 2:**
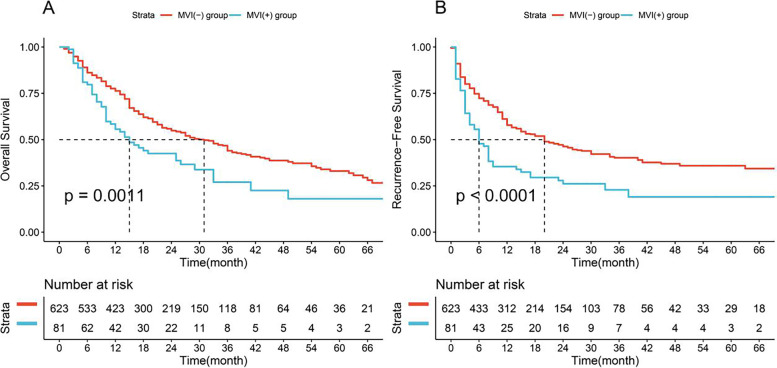
Comparison of survival rate between MVI-positive and negative groups before PSM. **A** Overall Survival; **B** Recurrence-Free Survival. MVI, microvascular invasion. PSM, Propensity Score Matching

All patients were followed up according to the standard procedure, and the periods ranged from 1 to 77 months. Before PSM, the median OS time was 12 months in the MVI-positive group and 17 months in the MVI-negative group. The median RFS time was 6 months for the MVI-positive group and 11 months for the MVI-negative group. The OS rates at 1, 3, and 5 years of the MVI-positive group were 55.6, 27.0, and 18.0%, respectively, and those of the MVI-negative group were 76.3, 44.0, and 33.0%, respectively. In addition, in the MVI-positive group, the RFS rates of ICC patients at 1, 3, and 5 years after curative resection were 35.9, 23.2, and 19.3%, respectively; and in the MVI negative group, these RFS rate were 57.8, 40.0, and 35.7%, respectively (Fig. 2A, B).

**Fig. 3:**
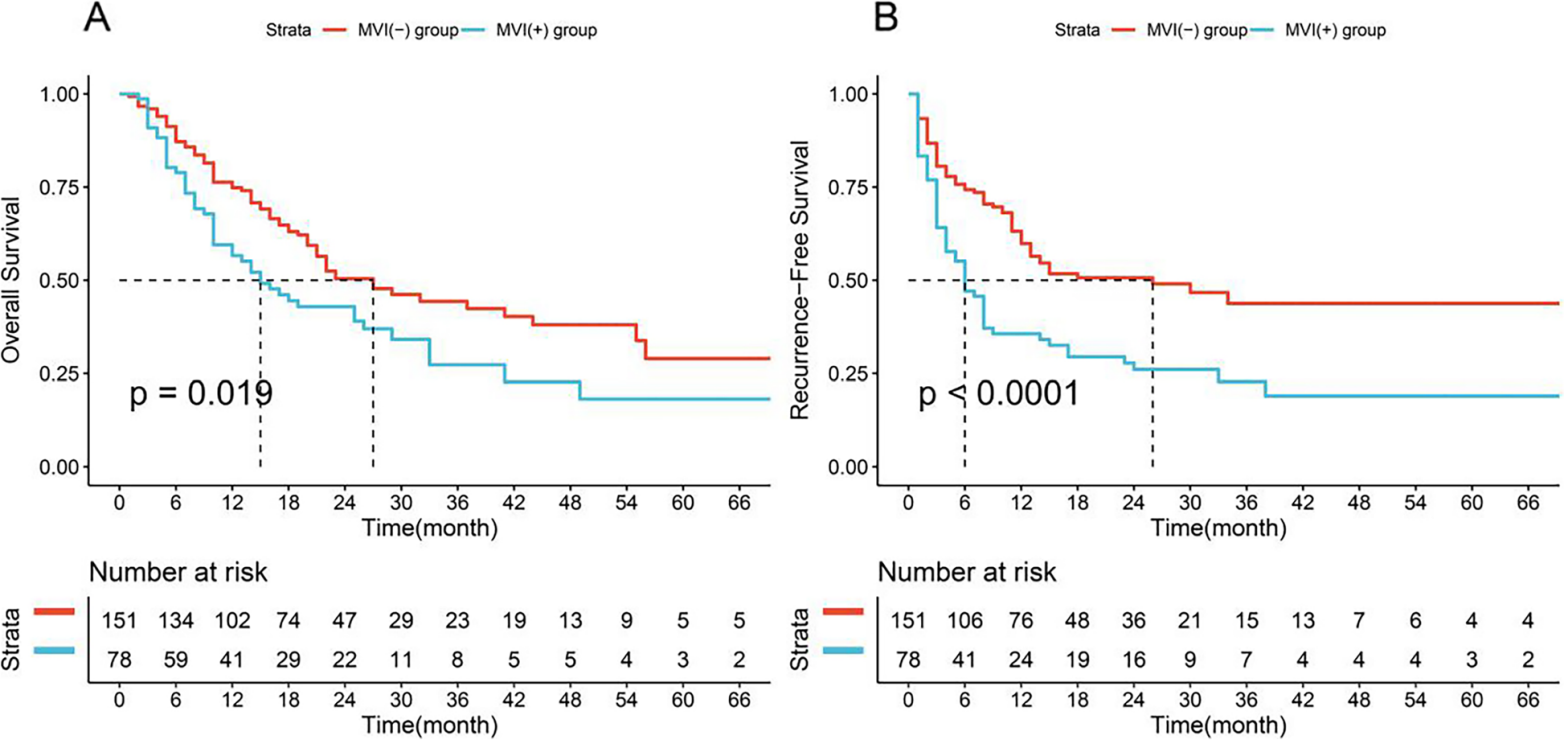
Comparison of survival rate between MVI-positive and negative groups after PSM. **A** Overall Survival; **B** Recurrence-Free Survival. MVI, microvascular invasion. PSM, Propensity Score Matching

After PSM, in the MVI-positive group, the median OS and RFS times were 12 and 5 months, respectively. In the MVI-negative group, the median OS and RFS times were 17 and 12 months, respectively. In addition, in the MVI-positive group, the OS and RFS rates at 1, 3, and 5 years were 56.6, 27.3, and 18.2% and 35.6, 22.8, and 19.0%, respectively. In the MVI-negative group, the OS and RFS rates at 1, 3, and 5 years were 74.8, 44.3, and 29.0% and 59.8, 43.8, and 43.8%, respectively (Fig. 3A, B).

There were significant differences in the OS (*p* = 0.001) and RFS (*p* < 0.001) curves between groups before PSM. Moreover, the OS (*p* = 0.002) and RFS (*p* < 0.001) curves between groups were also significantly different.

### MVI-related preoperative factors

**Fig. 4:**
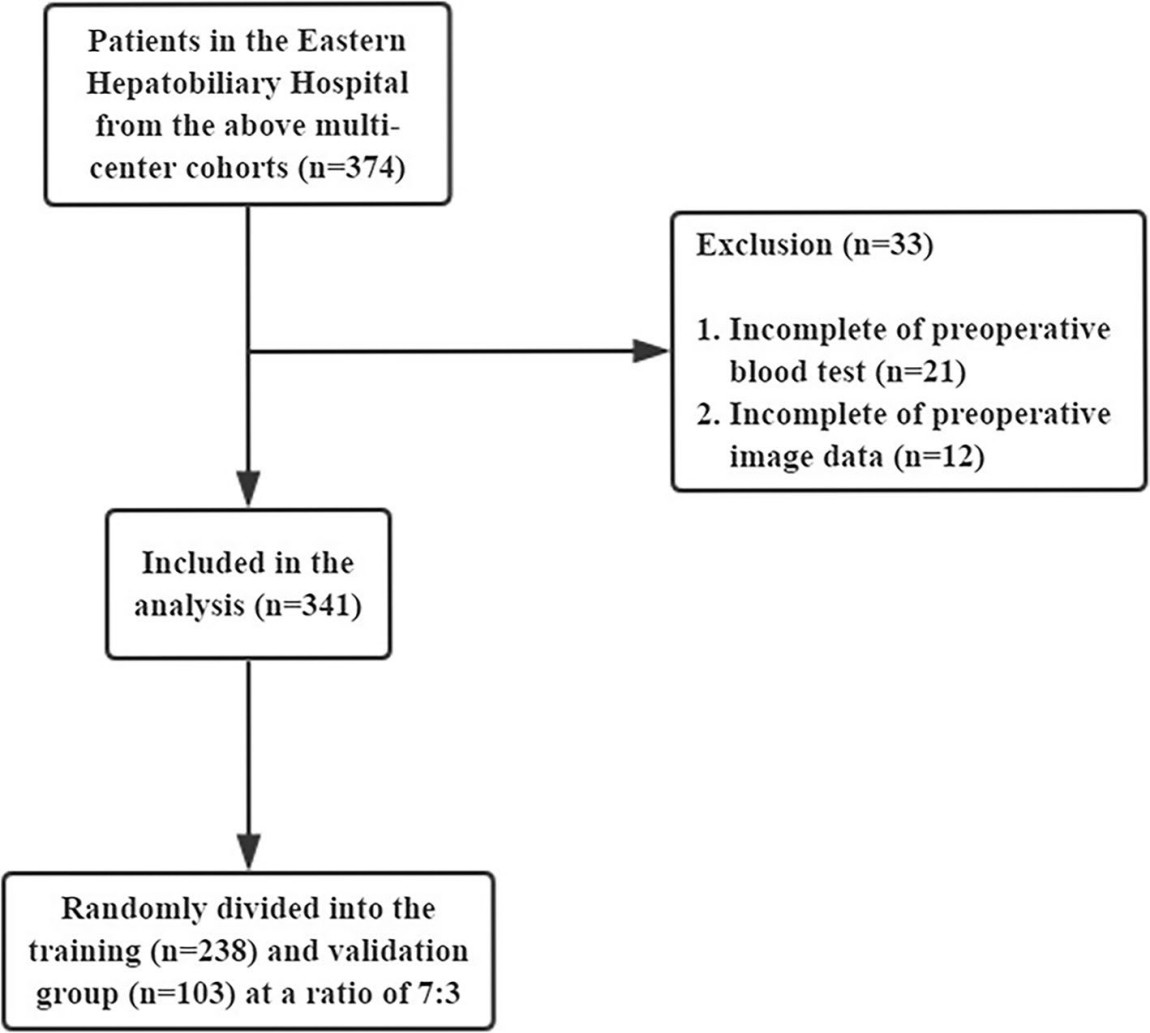
The flow chart of selected patients for constructing prediction model

A total of 374 patients from the Eastern Hepatobiliary Hospital in the above multicenter cohorts were initially included. According to the exclusion criteria, 341 patients were finally included for analysis and model construction. The cohort was randomly divided into a training group (238 patients) and a validation group (103 patients) at a ratio of 7:3 (Fig. 4).

Univariate and multivariate logistic regressions were adopted to analyze the correlation between preoperative factors and MVI in the training group. The results are shown in Table 7. Age (*p* = 0.049), GGT (*p* = 0.001), and preoperative tumor number (*p* = 0.004) were independent risk factors for the occurrence of MVI.

**Table 7:** Univariate and multivariate logistic regression for the occurrence of MVI in ICC patients

Variables	Univariate			Multivariate		
	OR	95% CI	***P***-value	OR	95% CI	***P-value***
**Age**	0.963	0.928–0.998	0.039	0.962	0.926–1.000	0.049
**Sex,** male vs female	1.054	0.478–2.321	0.897			
**Hepatitis**, no vs yes	0.676	0.312–1.463	0.320			
**White blood cell count**	1.112	0.929–1.331	0.246			
**Red blood cell count**	1.298	0.630–2.675	0.480			
**Hemoglobin**	0.997	0.973–1.021	0.791			
**Total bilirubin**	0.969	0.900–1.043	0.395			
**Albumin**	0.945	0.850–1.052	0.301			
**NLR**	1.244	0.958–1.615	0.101			
**PLR**	1.003	0.997–1.009	0.391			
**LMR**	1.035	0.881–1.217	0.676			
**GGT**	1.004	1.002–1.007	0.001	1.005	1.002–1.007	0.001
**AFP**	1.002	1.000–1.003	0.075			
**CA199**	1.001	0.999–1.002	0.332			
**CEA**	1.008	0.996–1.020	0.189			
**Image tumor diameter,** cm ≤5 vs > 5	1.434	0.643–3.200	0.379			
**Image tumor number,** single vs multiple	3.318	1.495–7.363	0.003	3.491	1.496–8.148	0.004

*Abbreviations*: *MVI* Microvascular invasion, *ICC* Intrahepatic cholangiocarcinoma, *OR* Odds ratio, *CI* Confidence interval, *NLR* Neutrophil-to-lymphocyte ratio, *PLR* Platelet-to-lymphocyte ratio, *LMR* Lymphocyte-to-monocyte ratio, *GGT* Gamma-glutamyl transpeptidase, *AFP* Alpha-fetoprotein, *CA199* Carbohydrate antigen 199, *CEA* Carcinoembryonic antigen

### Construction of a preoperative prediction model of MVI

**Fig. 5:**
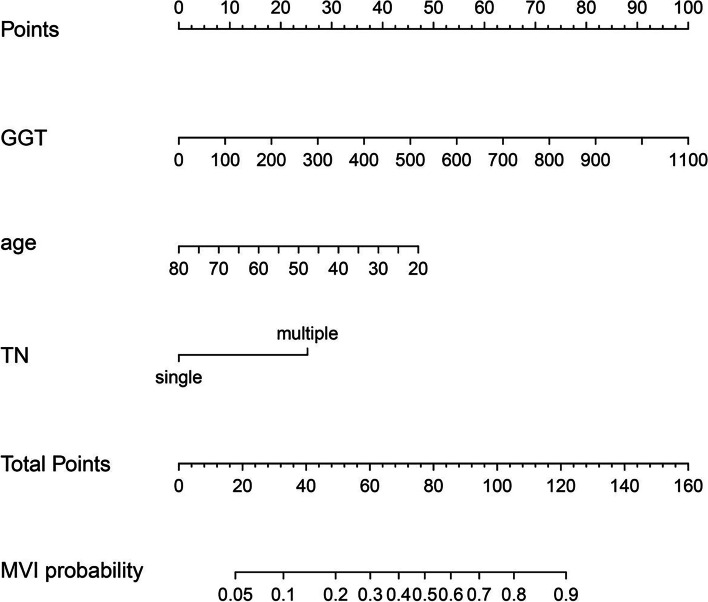
The nomogram of preoperative prediction model for occurrence of MVI. GGT, gamma-glutamyl transpeptidase. TN, preoperative image tumor number. MVI, microvascular invasion

Based on the above multivariate analysis results, age, GGT, and preoperative tumor number images were finally included to construct a preoperative prediction model for predicting the occurrence of MVI in ICC patients (Fig. 5).

**Fig. 6:**
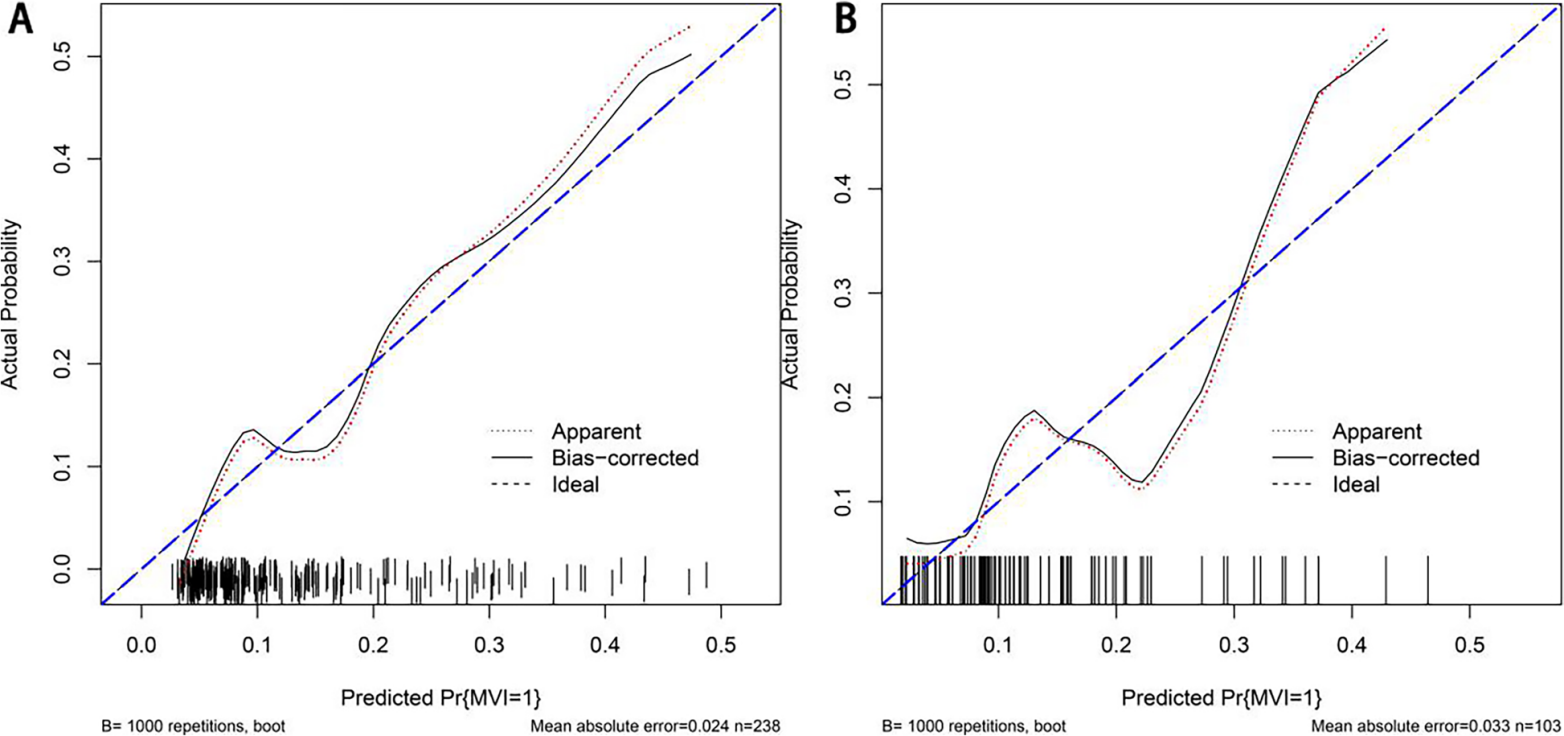
The calibration curve for the nomogram

The C-index was used to evaluate the predictive ability of the constructed nomogram. In the training and validation groups, the C-index was 0.7622 (95% CI: 0.6760–0.8484) and 0.7591 (95% CI: 0.6162–0.9020), respectively. The calibration curve demonstrated that the occurrence of MVI predicted by the prediction model was in accordance with the actual situation of MVI confirmed by postoperative pathology in the training and validation groups, respectively (Fig. 6A and B).

## Discussion

The early-stage clinical symptoms of ICC patients are often atypical and may manifest as abdominal pain, dyspepsia, weight loss, and abnormal liver function. Most patients are already in the advanced stage when they seek medical treatment. For advanced or unresectable cholangiocarcinoma, current adjuvant chemotherapy has limited effects. At present, gemcitabine combined with cisplatin is recommended as a first-line treatment; however, the median OS and RFS times are 11.7 and 8 months, respectively [21]. Surgical resection is still the only curative treatment [4]. This study was a multicenter retrospective study, and multivariate results indicated that the Child-Pugh grade, total bilirubin, CEA, major hepatectomy, pathological tumor diameter, satellites, surgical margin width, and MVI were independent risk factors for the prognosis of ICC patients with OS or RFS after curative resection. Among them, pathological tumor diameter is a well-known risk factor for prognosis in ICC patients, and it has also been included as an index for staging ICC in the 8th edition of the AJCC staging system [8]. The Child-Pugh grade has also been confirmed as a crucial factor for RFS in ICC patients after hepatectomy by Jeong S et al. [22] and subsequently has been included in the construction of a prediction model with good predictive performance. In addition, the surgical margin width, satellites, and high bilirubin levels have also been reported to be correlated with the prognosis of ICC patients [23–25]. The above previous research results are basically consistent with the results of our study. In addition, our study also found that CEA and major hepatectomy were independent risk factors for the prognosis of ICC patients after hepatectomy. Although the optimal cutoff value of CA199 was set to 200 U/ml based on the present research [26], the final results did not support it as an independent risk factor.

However, the prognostic value of MVI in ICC patients remains unclear. In the report of Jiao et al. [27] and Wang et al. [28], the relationship between MVI and ICC patients was not significant. HU et al. [14] showed that MVI was an important factor for RFS in ICC patients after surgery but not for OS. In the retrospective study of Chen et al. [29], MVI was a vital factor for OS but not RFS. Tang et al. [13] implemented PSM methods and demonstrated that MVI was a crucial factor for OS and RFS in ICC patients after surgery. Until now, relevant meta-analyses have not been reported to further clarify the relationship between them. The paper presents a multicenter retrospective study, and the results show that MVI is an independent risk factor for the prognosis of ICC patients after curative resection through the PSM method. The survival curves clearly showed that the prognoses in the MVI negative group were superior to the positive group with statistically significant.

Although the diagnosis of MVI was based on the pathology, a certain degree of systematic errors occurred due to the differences in diagnostic techniques and criteria. The incidence of MVI in ICC patients varies greatly among the existing related studies, ranging from 9.5 to 60.4% [13, 14, 26–30]; however, the incidence of MVI in this study was 11.5%, which was similar to the incidence of MVI in large-sample and multicenter studies. Therefore, this conclusion still needs to be further confirmed.

The status of MVI has a certain guiding value for the choice of the treatment strategy. A study that adopted PSM methods showed that in MVI-positive HCC patients, anatomical resection would be beneficial for improving the prognosis [31]. Another retrospective study has shown that wide surgical margins (≥1 cm) benefit survival in patients with MVI but not in patients without MVI [32]. Therefore, it is quite necessary to preoperatively predict the occurrence of MVI in ICC patients, and appropriate measures can be selected according to the predicted results to improve the prognosis of patients. At present, studies associated with preoperative prediction are rare. The prediction model established by Tang et al. [13] included tumor diameter, tumor capsule, AFP, and glutamic-pyruvic transaminase, and the C-index value of the training and validation groups were both approximately 0.7. Peng et al. [16] constructed a prediction model using ultrasound radiomics signatures, and the C-index values of the training and validation group were 0.699 and 0.756, respectively. An MRI-related study on ICC patients showed that some imaging features were correlated with the occurrence of MVI, but multivariate results did not suggest any factor was an independent risk factor [15]. Another related study that predicted MVI in patients with mass type ICC showed that large tumor diameter and high apparent diffusion coefficient (ADC) were independent risk factors for MVI, and the area under the receiver operating curve (AUC) was 0.782 when the ADC value was only used [17].

Our study determined that age, GGT, and preoperative image tumor number were independent risk factors for the occurrence of MVI. The prediction model based on the above factors showed good predictive ability and consistency. Unfortunately, due to data limitations, radiomics signatures could not be included in the prediction model. Including such data will definitely further improve the prediction ability.

These three factors (age, GGT, and preoperative image tumor number) have not been reported in other studies. However, they have been proven to be associated with the occurrence of MVI in HCC patients. Reports have indicated that young age is closely related to the occurrence of MVI and included this factor in the prediction models [33, 34]. Regarding GGT, Zhang et al. [32] and Zhao et al. [35] have shown that a high level of GGT is a vital factor for the occurrence of MVI in HCC patients. Wang et al. [36] found that tumor number was an independent risk factor for the occurrence of MVI in HCC patients. Although these indicators have been reported in the prediction model for HCC patients, this study included these factors for the first time in the prediction model for ICC patients.

Currently, the value of adjuvant therapy after curative resection in ICC patients is unclear. A meta-analysis [6] suggested that adjuvant postoperative radiotherapy and chemotherapy did not have any benefit for ICC patients; however, another meta-analysis [37] found that adjuvant therapy improved the prognosis of ICC patients after surgery. At the same time, a multicenter retrospective study [38] showed that adjuvant therapy was only beneficial for the “medium-risk group” and not for the “low-risk group” or the “high-risk group” after ICC surgery. Therefore, whether MVI could be combined with other indicators to distinguish ICC patients after surgery and select specific patients for adjuvant treatment remains to be further studied.

This study still had some limitations. First, although this study was a multicenter retrospective cohort study that adopted PSM method to reduce selective bias, systematic bias might still exist. Second, due to the long period of sample collection and multicentric data, the pathological diagnostic criteria for MVI might not be consistent. Third, the preoperative prediction model did not have an external validation group because of the lack of data.

In conclusion, a multicenter retrospective study or prospective study with a large sample is required to further confirm the vital value of MVI in the prognosis of ICC patients and to verify the prediction model proposed in this study.

## Conclusion

MVI is an independent risk factor for the prognosis of ICC patients after curative resection. Age, GGT, and preoperative image tumor number were independent risk factors for the occurrence of MVI in ICC patients. The constructed prediction model further showed good predictive ability in both the training and validation group, and the results were good consistent with reality.

## Data Availability

All data included in this study are available by contact with the corresponding author.
